# Tocilizumab for treating mevalonate kinase deficiency and TNF receptor-associated periodic syndrome: a case series and literature review

**DOI:** 10.1186/s12969-023-00952-2

**Published:** 2024-01-05

**Authors:** Yandie Li, Meiping Lu

**Affiliations:** Department of Rheumatology Immunology and Allergy, Children’s Hospital, Zhejiang University School of Medicine, National Clinical Research Center for Child Heath, NO.3333, Bin-sheng Road, 310052 Hangzhou, China

**Keywords:** Mevalonate kinase deficiency (MKD), TNF receptor-associated periodic syndrome (TRAPS), Tocilizumab, Treatment

## Abstract

**Background:**

Mevalonate kinase deficiency (MKD) and TNF receptor-associated periodic syndrome (TRAPS) are categorized as systemic autoinflammatory diseases (SAIDs), which are rare diseases characterized by early onset, severe conditions, and challenging diagnosis and treatment. Although different SAIDs have varying standard treatments, some SAIDs are poorly controlled after routine treatment, seriously affecting the growth and development of children and their quality of life. This study aims to provide more treatment strategies for SAIDs.

**Case presentation:**

We present two Chinese patients with MKD and TRAPS who were resistant to TNF- (tumor necrosis factor-) α blockade. After using etanercept, baricitinib, and glucocorticoid, patients with MKD and TRAPS still had periodic fever and rash. Due to the unavailability of IL-1 antagonists in the Chinese Mainland, we started administering intravenous tocilizumab (TCZ) at a dosage of 240 mg every three weeks. They had not experienced fever or rash after receiving one or two doses of TCZ. Before treatment with TCZ in the MKD patient, white blood cell (WBC) count, and TNF-α level were normal, erythrocyte sedimentation rate (ESR) and C-reactive protein (CRP) increased significantly, and IL-6 increased slightly. After treatment with TCZ, ESR and CRP levels returned to normal; however, IL-6 increased occasionally. In the TRAPS patient, ESR, CRP, WBC, IL-6, and TNF-α levels were increased significantly. After TCZ treatment, ESR, CRP, WBC, IL-6, and TNF-α levels returned to normal. The two patients were treated with TCZ for more than six months and achieved clinical and serological remission. Furthermore, they had no adverse reactions after injection of TCZ.

**Conclusion:**

In the absence of IL-1 antagonists in mainland China, tocilizumab emerges as an alternative drug in SAIDs that are resistant to TNF-α blockade.

**Supplementary Information:**

The online version contains supplementary material available at 10.1186/s12969-023-00952-2.

## Background

Systemic autoinflammatory diseases (SAIDs) are rare and severe diseases caused by gene mutations in innate immunity, leading to proinflammatory cytokine overproduction [1]. However, some SAIDs, such as systemic juvenile idiopathic arthritis (SJIA), are caused by multiple genes. Since Kastner first proposed the concept of SAIDs in 1999 [2], over 40 syndromes have been classified into this category [3]. SAIDs include mevalonate kinase deficiency (MKD) and TNF (tumor necrosis factor) receptor-associated periodic syndrome (TRAPS). After general treatment, some SAIDs remain challenging to control. This study aims to provide more treatment strategies for MKD and TRAPS.

MKD is an autosomal recessive disease caused by mevalonate kinase (MVK) in the cholesterol biosynthesis pathway. *MVK* mutation leads to pyrin inflammasome activation and increases the IL-1β secretion [4]. MKD symptoms include high fever, rash, splenomegaly, arthralgia, and psychomotor retardation [5]. In the acute phase, laboratory examination of MKD patients reveals leukocytosis, elevated C-reactive protein (CRP), and erythrocyte sedimentation rate (ESR). A serum immunoglobulin D (IgD) level greater than 100,000 U/L is considered a marker of MKD. However, 22% of MKD patients are reported to have normal IgD levels [6]. Moreover, MKD attacks can be triggered by a mild infection. Corticosteroids and anakinra are effective in reducing the severity of MKD. According to previous case reports, tocilizumab (TCZ) is also beneficial in treating MKD patients who are resistant to TNF-α blockade [7–14].

TRAPS is an autosomal dominant disease caused by heterozygous mutations in the TNF receptor superfamily 1 A (*TNFRSF1A*) gene encoding the TNF-α receptor. The accumulation of misfolded TNFR1 in the cytoplasm activates the nuclear factor- (NF-) κB and reactive oxygen species (ROS), resulting in proinflammatory cytokine IL-1β and TNF-α production [15]. The disease is characterized by unexplained fever, skin rash, serositis, arthralgia, and myalgia [16]. Symptom remission can be achieved with short-term corticosteroids and nonsteroidal anti-inflammatory drugs. Etanercept is considered an effective treatment option because TNF receptor abnormalities are linked to the pathology of TRAPS. Treating TRAPS with canakinumab and anakinra has resulted in high rates of clinical remission [17, 18]. In previous case reports, patients with TRAPS exhibited rapid improvement in clinical symptoms after receiving TCZ treatment [19].

TCZ is a humanized, monoclonal antibody against the IL-6 receptor (IL-6R), which can bind to soluble IL-6R, inhibiting IL-6 signaling [20]. SAIDs pathogenesis involves the abnormal activation of inflammasomes, resulting in IL-1β and IL-18 overproduction. Consequently, they stimulate inflammatory cytokine production, such as IL-6 and TNF-α [21]. Therefore, treatments such as TCZ have gained attention as potential treatments for SAIDs. Here, we report MKD and TRAPS cases that are resistant to TNF-α blockade and are successfully treated with TCZ. This case series can help expand the TCZ treatment database for MKD and TRAPS.

## Case presentations

### Patient 1

**Fig. 1:**
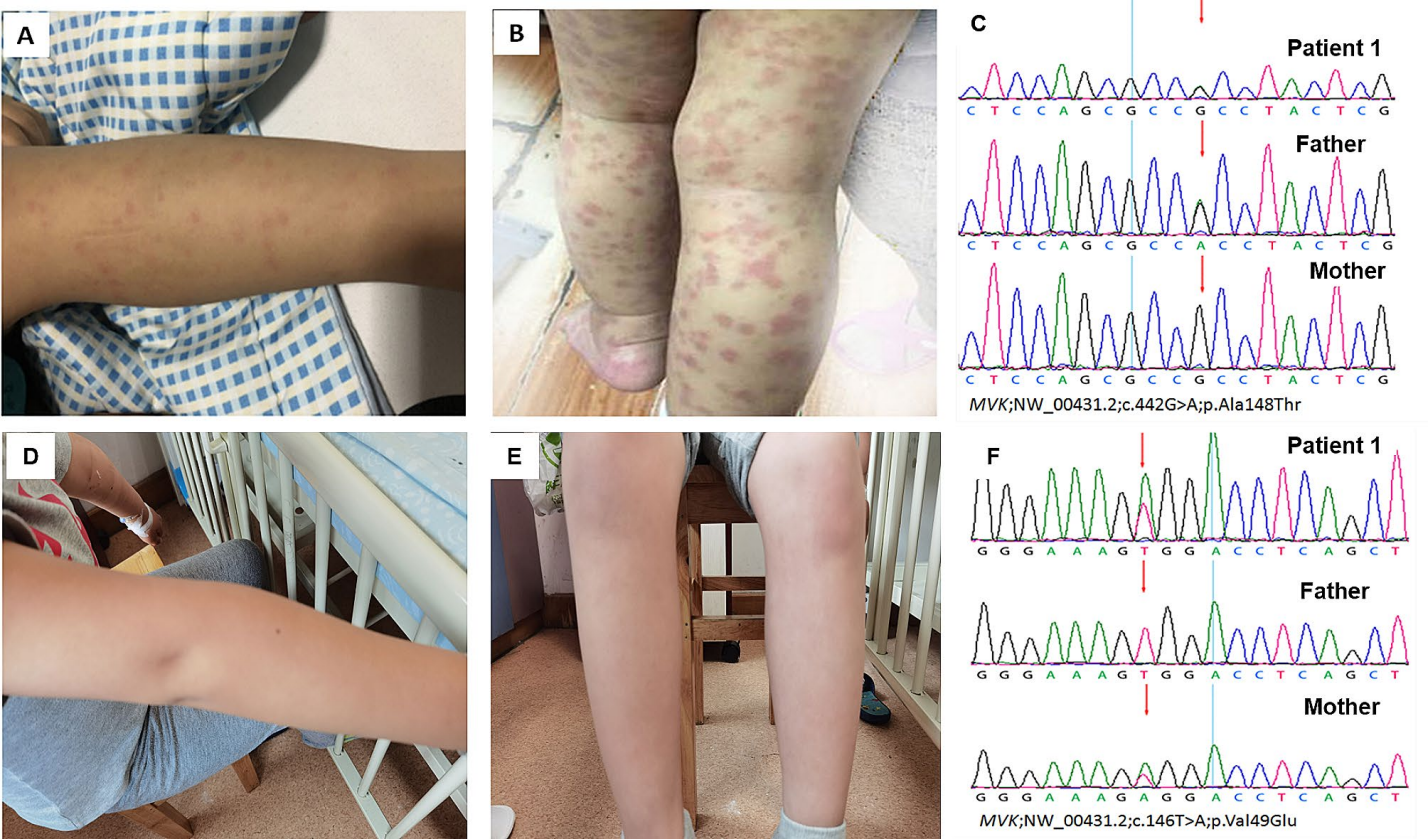
Skin rash and pedigrees with mutations in *MVK* leading to MKD. **(A**-**B)** The four limbs with an erythematous maculopapular rash before treatment. **(D**-**E)** The four limbs without rash after treatment of tocilizumab. **(C, F)** The pedigrees with mutations in MVK, which were compound heterozygous mutations (p.Ala148Thr; p.Val49Glu) inherited from mother and father, respectively

**Fig. 2:**
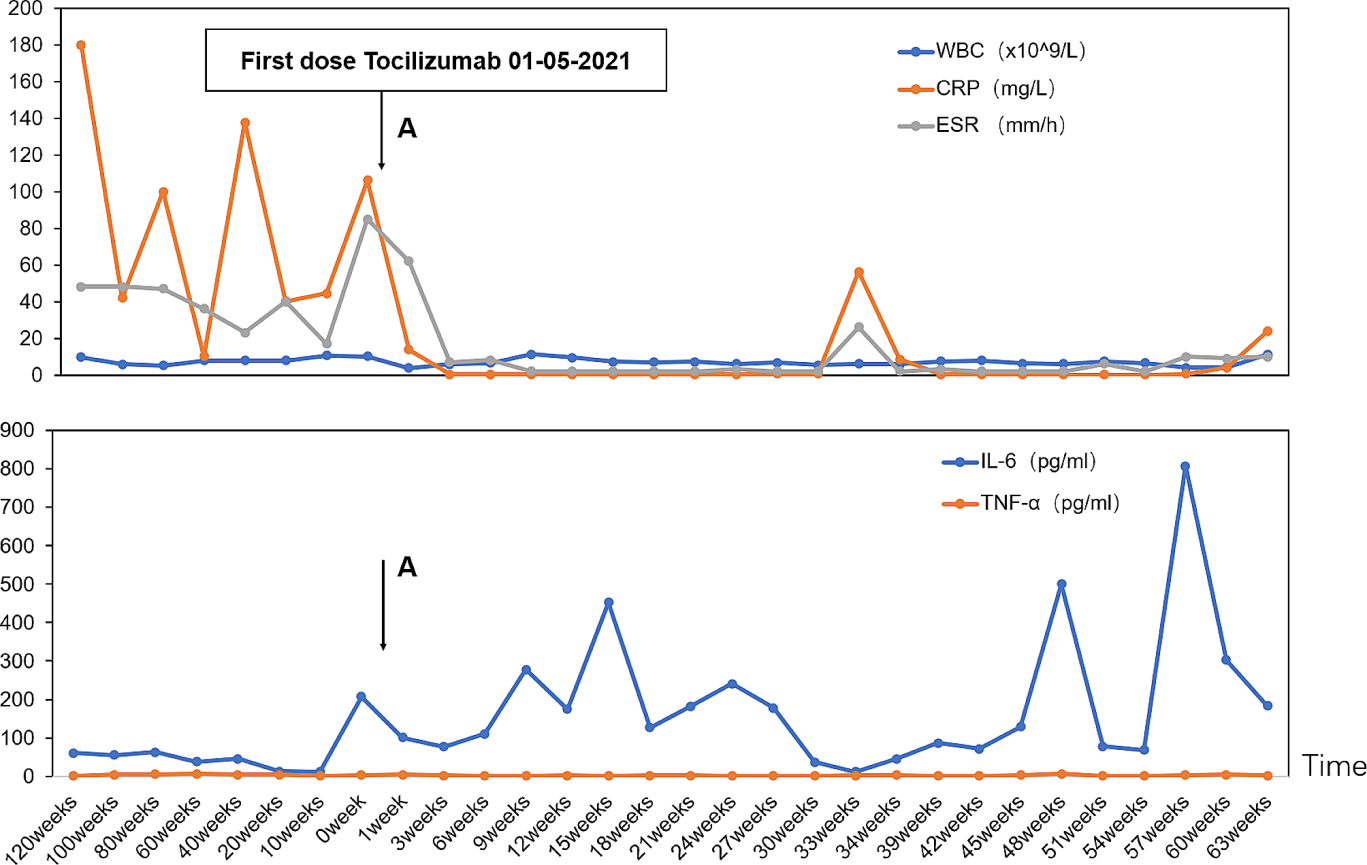
ESR, CRP, WBC, IL-6, and TNF-α levels before and after treatment of tocilizumab in the MKD patient. Arrow A indicates the first administration of tocilizumab (240 mg every three weeks). After treatment of tocilizumab in the MKD patient, ESR and CRP fell to normal; however, IL-6 increased occasionally. The levels of WBC and TNF-α were always in the normal range

A 3-year-and-11-month-old Chinese boy presented to the Children’s Hospital, Zhejiang University School of Medicine, on December 29, 2018, because he had been experiencing recurring fever and rash for over two years (Table 1). He had recurrent fever of more than 39 °C every 2–3 weeks, lasting 3–7 days. The erythematous maculopapular rash that covered the entire body appeared at high temperatures and faded when the body temperature dropped (Fig. 1A and B). There was no family history of diseases such as prolonged febrile illnesses or genetic disorders. On admission, physical examination revealed cervical lymph node enlargement and splenomegaly. No abnormality was found in other physical examinations. The laboratory indicators indicated that when the febrile episode flared up, CRP and ESR were significantly increased. Infections with Epstein–Barr virus, cytomegalovirus, and other viruses and tuberculosis were excluded. Concurrently, blood system diseases and solid tumors were excluded. No evidence of bacterial infection was found in the blood. Ultrasound suggested an enlargement of the liver, spleen, and superficial lymph nodes. Cardiac ultrasound did not indicate coronary artery dilation. He had no immune deficiency. For more than two years, the patient had intermittent oral mucositis. During this period, he was diagnosed with sepsis and/or Kawasaki disease. After treatment with antibiotics, intravenous immunoglobulin, and even glucocorticoids, the child’s clinical symptoms improved, and his inflammatory biomarkers returned to normal. However, the child still experienced recurrent fever and rash. Finally, we recommended that the patient and his parents undergo genetic testing. In the patient, two pathogenic mutations were detected in the *MVK* gene (NM_000431.2): c.442G > A, p.Ala148Thr (A148T) and c.146T > A, p.Val49Glu (V49E) **(**Fig. 1C and F**)**. In the acute phase of this patient’s condition, the IgD level in serum was 200 mg/L, and the MKD activity was 17.9 µg/L. The normal range for serum IgD is 10-40 mg/L. The normal reference value range of plasma MVK activity of Chinese healthy children is 96.17 ± 19.24 µg/L. Unfortunately, we did not measure mevalonic acid levels in urine. The patient was diagnosed with MKD based on his clinical manifestations, laboratory examination, and genetic results. Unfortunately, IL-1 antagonists were unavailable in the Chinese Mainland, so we empirically treated him with etanercept (0.8 mg/kg, subcutaneous injection once a week) for five months. After treatment of etanercept, he still had recurrent fever, with the rare occurrence of rash. Then, he was given 1 mg of baricitinib a day for a month, which was ineffective. When the temperature could not be controlled, we administered glucocorticoids; however, they had significant side effects and could not be used for long. The patient experienced fever once a month that lasted for 7–10 days. On May 1, 2021, the patient began taking TCZ (Actemra, 80 mg/4 mL). There was no specific dose of tocilizumab used to treat MKD. We referred to TCZ doses used in treating SJIA and polyarthritis-JIA [22] in previous case reports on TCZ treatment of MKD. Finally, we injected a dose of 12 mg/kg every three weeks. After using TCZ, the temperature returned to normal, and the rash subsided gradually **(**Fig. 1D and E**)**. CRP and ESR returned to normal after six weeks. However, serum IL-6 level increased occasionally **(**Fig. 2**)**. At present, the patient’s symptoms are well controlled, and his weight has increased physiologically. TCZ administration resulted in an adenovirus infection with liver function damage. After the treatment, the body temperature and liver function returned to normal. There were no severe adverse reactions from the use of TCZ.

**Table 1:** Clinical characteristics of two patients

	Patient 1	Patient 2	References
Date of admission	2018/12/29	2018/8/2	
Age	3Y11M	7Y10M	
Gender	M	M	
Blood routine			
WBC counts (10^9^/L)	9.68	23.34	4–12.0
PMNs (%)	64.5	86.2	50–75
Hgb (g/L)	101	93	110–155
PLT (10^9^/L)	151	463	100–400
CRP (mg/L)	179.79	183.57	0–8
ESR (mm/h)	48	> 140	0–20
Alb(g/L)	39.1	31.1	32–52
ALT(U/L)	9	8	< 50
AST(U/L)	29	12	15–60
Serum ferritin(µg/L)	218.8	126.2	24–336
triglyceride(mmol/L)	2.15	0.67	< 1.7
LDH(U/L)	352	253	110–295
BUN(mmol/L)	2.86	2.69	1.79–6.43
Creatinine(µmol/L)	45	53	15–77
ASO	1.7	39	0-156
RF (U/ml)	3.1	2.7	0–30
Fibrinogen(g/L)	3.43	5.72	1.8-4
D-dimer(mg/L)	1.24	0.35	< 0.55
IL-2 (pg/ml)	1	1.5	1.1–9.8
IL-4(pg/ml)	1.6	2.3	0.1-3
IL-6 (pg/ml)	60.7	157.8	1.7–16.6
IL-10(pg/ml)	7.6	6.1	2.6–4.9
TNF-α(pg/ml)	1	64.9	0.1–5.2
IFN-γ(pg/ml)	35.3	1.2	1.6–17.3
IgG (g/L)	9.2	17.9	5-10.6
IgG1(g/L)	8.43	/	4.9–11.4
IgG2(g/L)	0.87	/	1.5–6.4
IgG3n (g/L)	0.17	/	0.2–1.1
IgG4n (g/L)	0.16	/	0.08–1.4
IgA (g/L)	4.81	2.45	0.34–1.38
IgM (g/L)	0.54	1	0.44–1.44
IgE (IU/mL)	103	344	< 100
C3 (g/L)	1.75	1.75	0.5–1.5
C4 (g/L)	0.48	0.38	0.1–0.4
CD 19(%)	25.5	19.6	18.5–28
CD3 (%)	68.5	70.7	56–68
CD4 (%)	41.8	44.2	29–40
CD8 (%)	20.4	22.9	19–25
CD3-CD16 + CD56+ (%)	5.9	0.4	9.0–19
CD4/CD8	2.05	1.93	1.1-2
ANA	negative	negative	< 1:80
ANCA	negative	negative	
EBVEA-IgM	0.01	0.06	< 1.1
EBVCA-IgM	1.63	1.39	< 40
MP-IgM	0.43	0.02	< 1.1
CP-IgM	0.01	0.06	< 1.1
TSPOT	negative	negative	
CMV IgM	/	/	

WBC: white blood cell; PMN: polymorphonuclear neutrophil; PLT: platelets; CRP: C reactive protein; ESR: erythrocyte sedimentation rate; Alb: albumin; ALT: alanine transaminase; AST: aspartate aminotransferase; LDH: lactic dehydrogenase; BUN: blood urine nitrogen; ASO: antistreptolysin; RF: rheumatoid factor; ANA: antinuclear antibody; ANCA: anti-neutrophil cytoplasmic antibody; EBVEA: Epstain-Barr virus early antigen; EBVCA: Epstain-Barr virus capsid antigen; MP: mycoplasma; CP: Chlamydia; TSPOT: T cell spot test; CMV: cytomegalovirus

### Patient 2

**Fig. 3:**
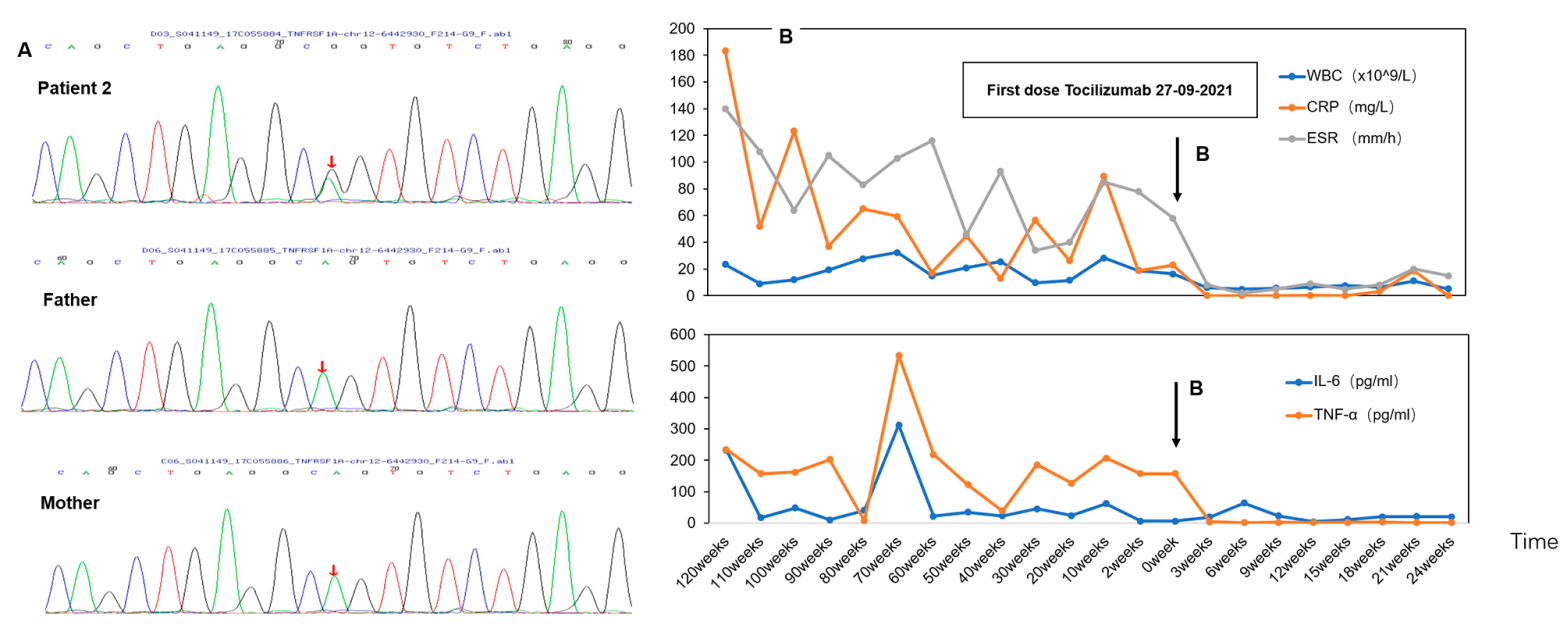
**(A)** The mutation in the *TNFRSF1A* gene (NM 0010 65; exon3): c.295T > C, p.Cys99Arg, which was a spontaneous mutation. **(B)** ESR, CRP, WBC, IL-6, and TNF-α levels before and after treatment of tocilizumab. Arrow B indicates the first administration of tocilizumab (240 mg every three weeks). After treatment of tocilizumab, ESR, CRP, WBC, IL-6, and TNF-α return to normal in the TRAPS patient

On August 2, 2018, a 7-year-and-10-month-old Chinese boy with no family history of febrile episodes was admitted to our department with a four-year history of recurrent fever (Table 1). The child had a fever once a month, lasting 10–14 days each time. Physical examination revealed cervical lymphadenectasis. The laboratory indicators indicated that WBC, CRP, ESR, IL-6, and TNF-α were significantly increased. The virus infections, tuberculosis, and tumors were excluded. No bacterium was found in the blood. Ultrasound suggested the enlargement of superficial lymph nodes. Cardiac ultrasound did not indicate coronary artery dilation. He has been previously diagnosed with sepsis. Following antibiotic treatment, the child’s body temperature and inflammatory indicators would return. However, the child still had recurrent fever. In this patient, one pathogenic mutation was detected in the *TNFRSF1A* gene (NM 0010 65; exon3): c.295T > C, p.Cys99Arg (Fig. 3A). Based on his clinical manifestations and the genetic results, he was diagnosed with TRAPS. When symptoms could not be controlled, he took glucocorticoids, but his parents refused to use glucocorticoids for a long time. This patient was treated with etanercept (0.8 mg/kg, subcutaneous injection once a week) for three years because of the absence of IL-1 antagonist agents. However, recurrent fever occurred every three months, lasting 10 days. On September 27, 2021, the boy began to take TCZ (dose: injection of 8 mg/kg once every three weeks). After three weeks of using TCZ, the temperature returned to normal, the rash subsided, and CRP, ESR, TNF-α, WBC, and IL-6 levels returned to normal (Fig. 3B). No adverse reactions occurred.

This study was approved by the Research Ethical Committee of the Children’s Hospital, Zhejiang University School of Medicine (2020-IRB-028). The patient’s parents provided informed consent for the collection and publication of data and photos.

### Literature review

Literature reviews were performed using “PubMed” and “Web of Science” by searching for the terms “Mevalonate kinase deficiency” and “TNF receptor-associated periodic syndrome” and “tocilizumab”. Table 2 [7–14] and 3 [23–26] summarize previously reported cases of using TCZ in treating MKD and TRAPS patients. In previous studies, a total of 11 MKD patients and 4 TRAPS patients were treated with TCZ. These patients received treatment with other biological agents, but their conditions were not adequately controlled. After using TCZ, most patients achieved complete remission.

**Table 2:** Summary the treatment of tocilizumab for MKD

case	1	2	3	4	5	6	7	8	9	10	11
Ancestry	Faroe Islands	ND	ND	ND	N. European	N. European	North African	Northern European	South Asian	European	European
Age	12 Y	36 Y	13 Y	ND	28Y	24Y	33Y	25Y	ND	18Y	43Y
Gender	F	F	F	ND	M	M	M	F	M	ND	M
Onset of symptoms	3 months	23 Y	infancy	ND	< 1Y	< 1Y	1y	< 1Y	ND	4Y	2Y
Gene mutation	p. V377I/c.417insC	p. V377I/c.417insC	p.V377l and p.H380R	p.V377I/I268T	ND	ND	p.N205D and p.G336S	p.I268T/V377I	p.S52N/D386N	p.V377I/I268T	p.V377I/L234P
Clinical phenotype	recurrent episodes of fever, pallor, fatigue, lymphadenopathy, abdominal pain, oral ulceration, arthralgia/myalgia of the lower limbs	episodic fever, abdominal pain, lymphadenopathy, hepatosplenomegaly	fever, headache, mouth ulcers, arthralgia, abdominal pain, cervical lymphadenopathy	ND	ND	ND	fever, abdominal pain, vomiting, otitis, odynophagia episodes, proteinuria, AA amyloidosis	fever, lymphadenopathy, gastrointestinal disturbance, rash, joint pain, sore throat	ND	ND	ND
serum IgD level	ND	750 kU/L	198,000 U/L	ND	ND	ND	ND	ND	ND	ND	ND
MK activity	ND	0 pmol/min/mg	ND	ND	ND	ND	ND	ND	ND	ND	ND
urine mevalonic acid/creatinine ratio	ND	3.0 mmol/moL	ND	ND	ND	ND	ND	ND	ND	ND	ND
Treatment prior to TCZ	Etanercept, anakinra	NSAIDs, simvastatin, anakinra	colchicine, corticosteroids, etanercept, anakinra	anakinra	NSAIDs, anakinra, etanercept	NSAIDs, anakinra	Colchicine	IL-1 blockade, etanercept	anakinra	anakinra, etanercept	anakinra, etanercept
TCZ dose (mg/kg) and route of administration	8 IV	8 IV	8 IV, 4 IV, 7 IV	8 IV	8 IV	8 IV	ND	ND	ND	ND	ND
Frequency of administration (weeks)	2	4	4	4	4	4	ND	ND	ND	ND	ND
Duration of treatment (months)	> 24 months	5 years	20 months	5 months	24 months	13 months	ND	ND	ND	ND	ND
Adverse events	NO	ND	recurrent upper respiratory tract infections (URTIs)	ND	ND	ND	ND	ND	ND	ND	ND
Outcome Clinical	CR	PR	CR(CR at dose of 8 mg/kg but due to adverse events dose reduced, ultimately with stable clinical and serological status on 7 mg/kg IV every 4 weeks)	CR	CR	CR	CR	CR	PR	CR	PR
Reference	Rafiq et al.(2018) [7]	Musters et al.(2015) [8]	Shendi et al.(2014) [9]	Stoffels et al.(2015) [10]	Lane et al.(2015) [11]	Lane et al.(2015) [11]	Rodrigues et al.(2020) [12]	Lane et al.(2013) [13]	Lane et al.(2013) [13]	ter Haar et al.(2016) [14]	ter Haar et al.(2016) [14]

F, female;Het, heterozygous; M, male;ND, not described;Y, years. CR: complete response; PR: partial response; TCZ: tocilizumab

**Table 3:** Summary the treatment of tocilizumab for TRAPS

case	1	2	3	4
Ancestry	ND	ND	ND	ND
Age	52Y	49Y	30Y	6Y
Gender	M	M	F	M
Onset of symptoms	5Y	ND	7Y	4Y
Gene mutation	C33Y	R92Q	no identified variant	C96R
Clinical phenotype	recurrent fevers, myalgia, rash, abdominal pains, joint pains, and lymphadenopathy	fever, recurrent annular erythematous plaques, conjunctivitis	high fever, nausea, rashes, migratory myalgia, joint pain	fever, arthritis, skin rash, vomiting, diarrhea, unilateral periorbital edema
Treatment prior to TCZ	corticosteroids, etanercept, anakinra	methotrexate, salazopyrin, hydroxychloroquine, infliximab, etanercept	prednisolone, etanercept, colchicine,	indomethacin, ibuprofen, hydroxychlorochine, cyclosporine A, methotrexate, infliximab
TCZ dose (mg/kg) and route of administration	8 IV	8 IV	8 IV	8 IV
Frequency of administration (weeks)	4	4	every 2 weeks then PRN	4
Duration of treatment (months)	6months	6months	26months	42months
Adverse events	Trombocytopenia	ND	ND	ND
Outcome Clinical	CR	CR	CR	CR
Reference	Vaitla et al. (2011) [23]	Akasbi et al.(2015) [24]	Hosoya et al. (2015) [25]	Torre et al. (2015) [26]

F, female;Het, heterozygous; M, male;ND, not described;Y, years. CR: complete response; PR: partial response; TCZ: tocilizumab

## Discussion

Many studies demonstrated that MKD and TRAPS are multicytokine-driven diseases, with the involvement of proinflammatory cytokines, including IL-1, IL-6, and TNF-α [27]. The most used biological agents of MKD and TRAPS are etanercept, canakinumab, and anakinra [28]. The 2021 EULAR/American College of Rheumatology guidelines recommend canakinumab as the first-line treatment for MKD and TRAPS [29]. However, the clinical symptoms and laboratory markers of our two patients did not improve after using etanercept, and they suffered from long-term pain. Unfortunately, there are no IL-1-related biological agents in the Chinese Mainland. From the literature review, we discovered that TCZ treatment was effective in MKD and TRAPS patients who were treated unsuccessfully with TNF-α blockade and IL-1 antagonists. Our two patients, MKD and TRAPS, who are resistant to etanercept, were treated with TCZ with clinical and serological remission.

Macrophages of TNFR1-mutant mice produce more IL-6 in response to lipopolysaccharides than wild-type macrophages [30]. IL-6 can trigger ROS production in monocytes from TRAPS patients [31]. IL-6 production decreases after ROS inhibition. Therefore, tocilizumab can interrupt the mechanism between IL-6 and ROS [15]. However, other studies demonstrated that cytokines (including TNF, IL-1, and IL-6) did not decrease after TCZ administration, thus suggesting that IL-6 inhibition may not affect TRAPS pathogenesis [23]. IL-6 is a crucial cytokine for plasma cell survival and may be involved in basophil differentiation. IgD also stimulates IL-6 release by prebasophil cells. The IL-6/IL-6 receptor and IgD pathways may be dysregulated in MKD [32]. Stimulation with TLR2 and NOD2 ligands increases IL-1a, IL-1β, IL-6, and TNF secretion in peripheral blood mononuclear cells with MKD patients. In vitro and in vivo, anakinra inhibit not only IL-1β (and IL-1a) but also IL-6, indicating a driving role for IL-1 [10]. However, some MKD patients do not achieve remission after anakinra treatment. TCZ induces remission and inhibits IL-1β production more than five times in some MKD patients. Therefore, MKD is considered to be a multicytokine disease [10]. In our study, patients with MKD occasionally had elevated IL-6, so we should be wary of disease recurrence. This increase in IL-6 levels may be related to the TCZ mechanism. TCZ can competitively bind to IL-6 receptors with IL-6, resulting in an increased level of free IL-6 [23].

TCZ has been found to be effective in treating rheumatoid arthritis, JIA, adult-onset Still diseases (AOSD), giant cell arteritis, cytokine release syndrome associated with tumor-specific T cell infusion therapy, and coronavirus 2019 (COVID-19). TCZ was administered subcutaneously or intravenously, either as monotherapy or in combination with disease-modifying anti-rheumatic drugs, which enables control of disease activity and normalization of serum inflammatory markers in both systemic and chronic articular forms of AOSD [33]. In a case-control study, TCZ treatment resulted in a significant decrease in the prednisolone dose, ESR, leucocyte count, CRP, and ferritin levels, and improvement in all clinical manifestations. Thus, TCZ is an effective and well-tolerated treatment option for drug-resistant AOSD that contributes to glucocorticoid sparing [34].

Like other immunosuppressants, TCZ treatment increases the risk of infection. TCZ can cause neutropenia, thrombocytopenia, elevated blood lipids, liver dysfunction, and infusion reactions. If the patient has severe or frequent infections, elevated liver enzymes or bilirubin, neutropenia, or thrombocytopenia, the TCZ dose is recommended to be reduced by 50% [35]. Dose adjustment may need to be individualized, and the TCZ dose may need to be increased as treatment time is extended. In our two patients, there were no adverse reactions after administering TCZ. However, recent data reveal that ESR and CRP of MKD patients have an upward trend. If the disease relapses, it may be necessary to increase the dose of tocilizumab and/or reduce administration time. However, current literature on the role of TCZ in TRAPS and MKD is limited. Therefore, we still need to be cautious when using TCZ to treat MKD and TRAPS.

## Conclusion

In this case series, we report the use of TCZ in treating an MKD patient and a TRAPS patient. TCZ may be an option for treating rare monogenic SAIDs. Further studies are warranted to determine the optimal dosage of TCZ. Longer follow-up is required to evaluate the long-term safety and efficacy of TCZ in treating MKD and TRAPS.

### Electronic supplementary material

Below is the link to the electronic supplementary material.

Supplementary Material 1

Supplementary Material 2

Supplementary Material 3

Supplementary Material 4

Supplementary Material 5

## Data Availability

The datasets used and/or analyzed during the current study are available from the corresponding author upon reasonable request.

## Abbreviations

MKD: Mevalonate kinase deficiency
TRAPS: TNF receptor-associated periodic syndrome
SAIDs: systemic autoinflammatory diseases
TNFRSF1A: TNF receptor superfamily 1 A
NSAIDs: Nonsteroidal anti-inflammatory drugs
AOSD: Adult-onset Still’s diseases
