# Supplementary figures and images for: Tocilizumab for treating mevalonate kinase deficiency and TNF receptor-associated periodic syndrome: a case series and literature review
Source: Pediatr Rheumatol Online J. 2024 Jan 5;22:11. doi: 10.1186/s12969-023-00952-2 (PMC10768362; doi:10.1186/s12969-023-00952-2)

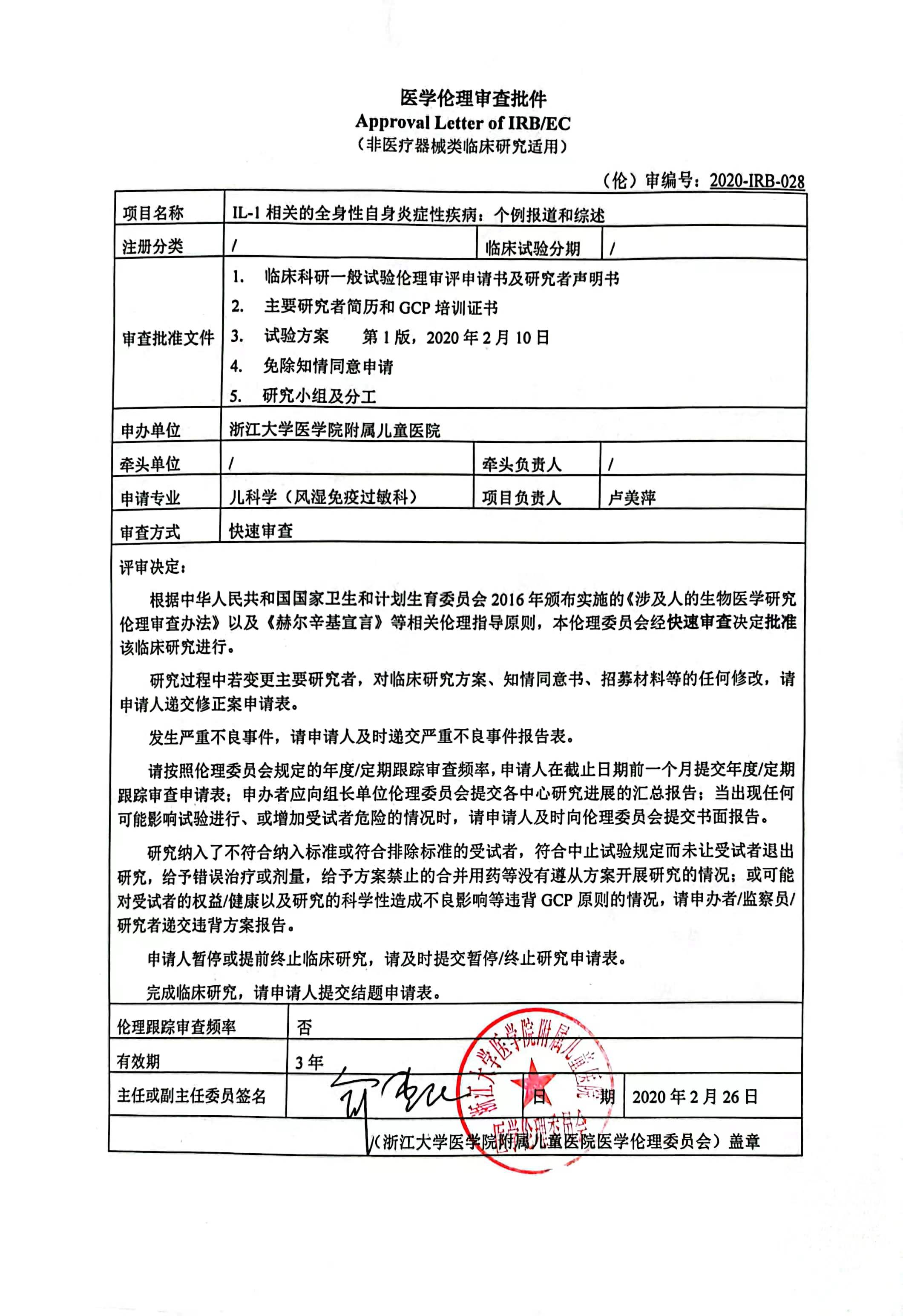

Supplement: Supplementary file 1 — Supplementary Material 1 [file 12969_2023_952_MOESM1_ESM.jpg]
