# Supplementary material for: Tocilizumab for treating mevalonate kinase deficiency and TNF receptor-associated periodic syndrome: a case series and literature review
Source: Pediatr Rheumatol Online J. 2024 Jan 5;22:11. doi: 10.1186/s12969-023-00952-2 (PMC10768362; doi:10.1186/s12969-023-00952-2)
Supplement: Supplementary file 3 — Supplementary Material 3 [file 12969_2023_952_MOESM3_ESM.pdf]

EDITORIAL CERTIFICATE

This certificate is issued as a confirmation that the paper mentioned below has been proofread and corrected by the HOME for Researchers' editorial team. After being checked and amended as seen appropriate, we feel that the standard of English in this manuscript satisfies the requirements of submission to journals to be considered for publication.

Manuscript Title

《Tocilizumab for Treating Mevalonate Kinase Deficiency and TNF Receptor-associated Periodic Syndrome: A Case Series and Literature Review》

Certificate Number

20230000260

Date Issued

2023-12-15

HOME for Researchers
