# Supplementary material for: Tocilizumab for treating mevalonate kinase deficiency and TNF receptor-associated periodic syndrome: a case series and literature review
Source: Pediatr Rheumatol Online J. 2024 Jan 5;22:11. doi: 10.1186/s12969-023-00952-2 (PMC10768362; doi:10.1186/s12969-023-00952-2)
Supplement: Supplementary file 4 — Supplementary Material 4 [file 12969_2023_952_MOESM4_ESM.pptx]

## Slide 1
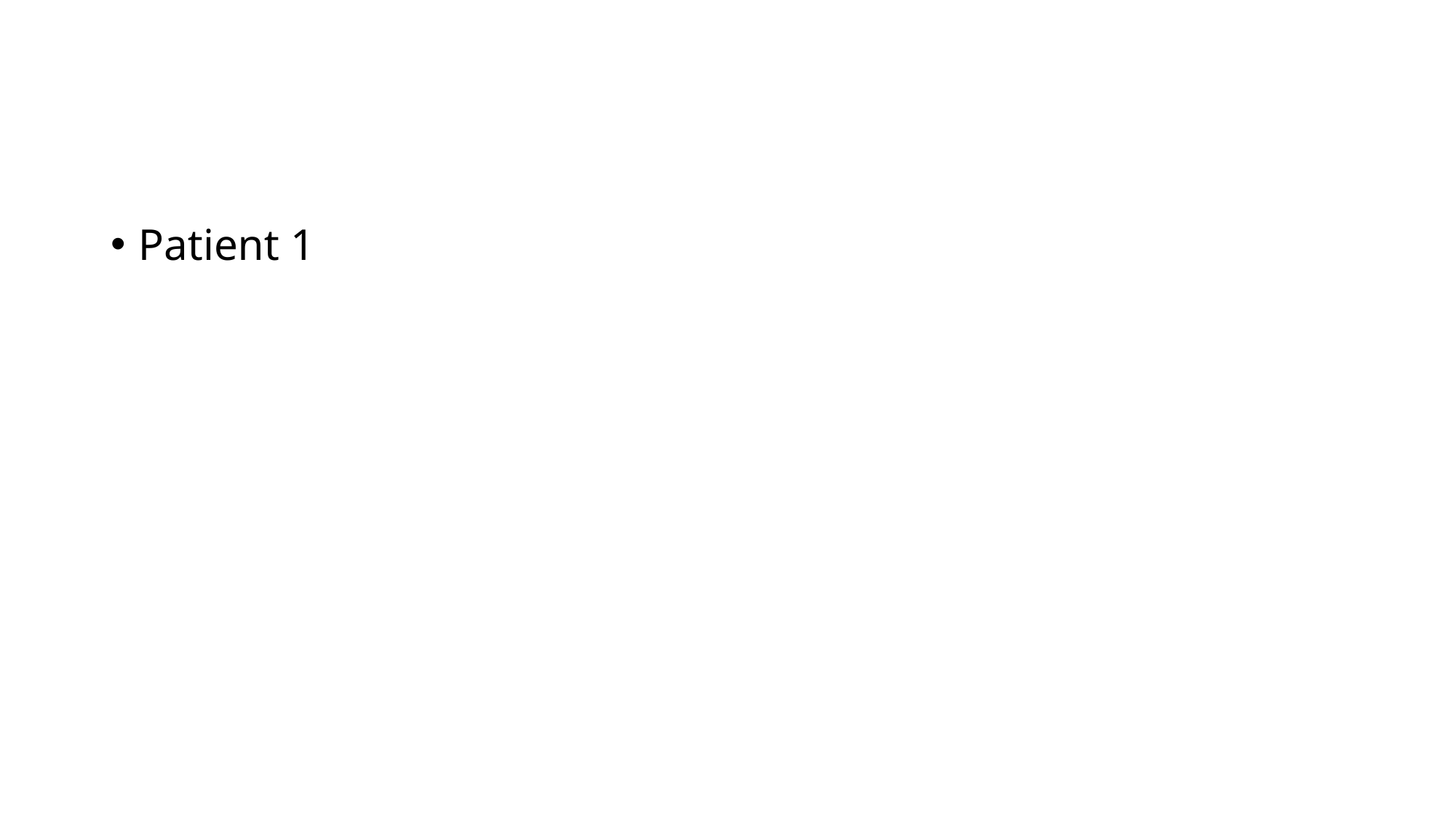

#
Patient 1

## Slide 2
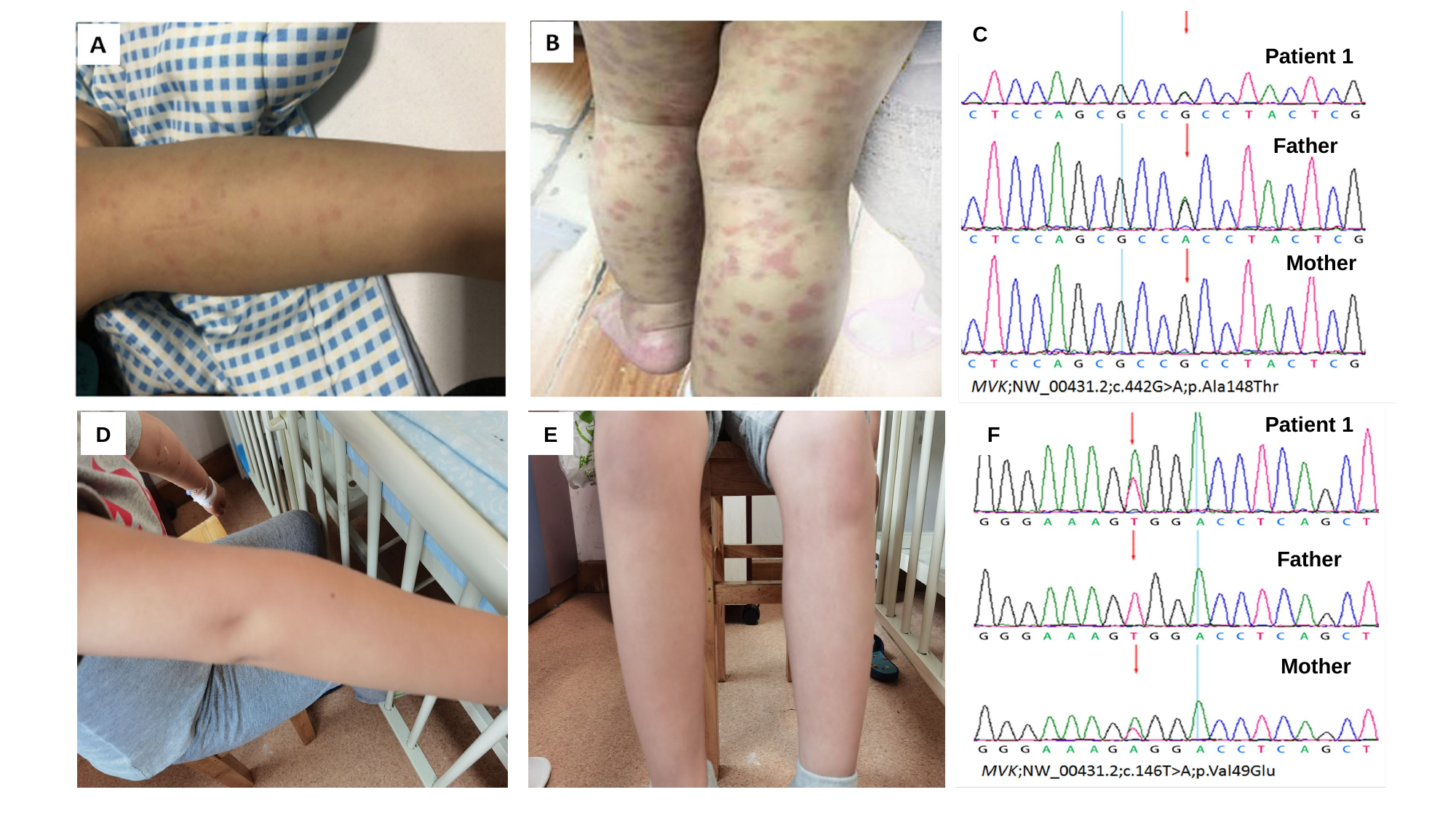

C
Patient 1
Father
Mother
D
E
F
Patient 1
Father
Mother

## Slide 3
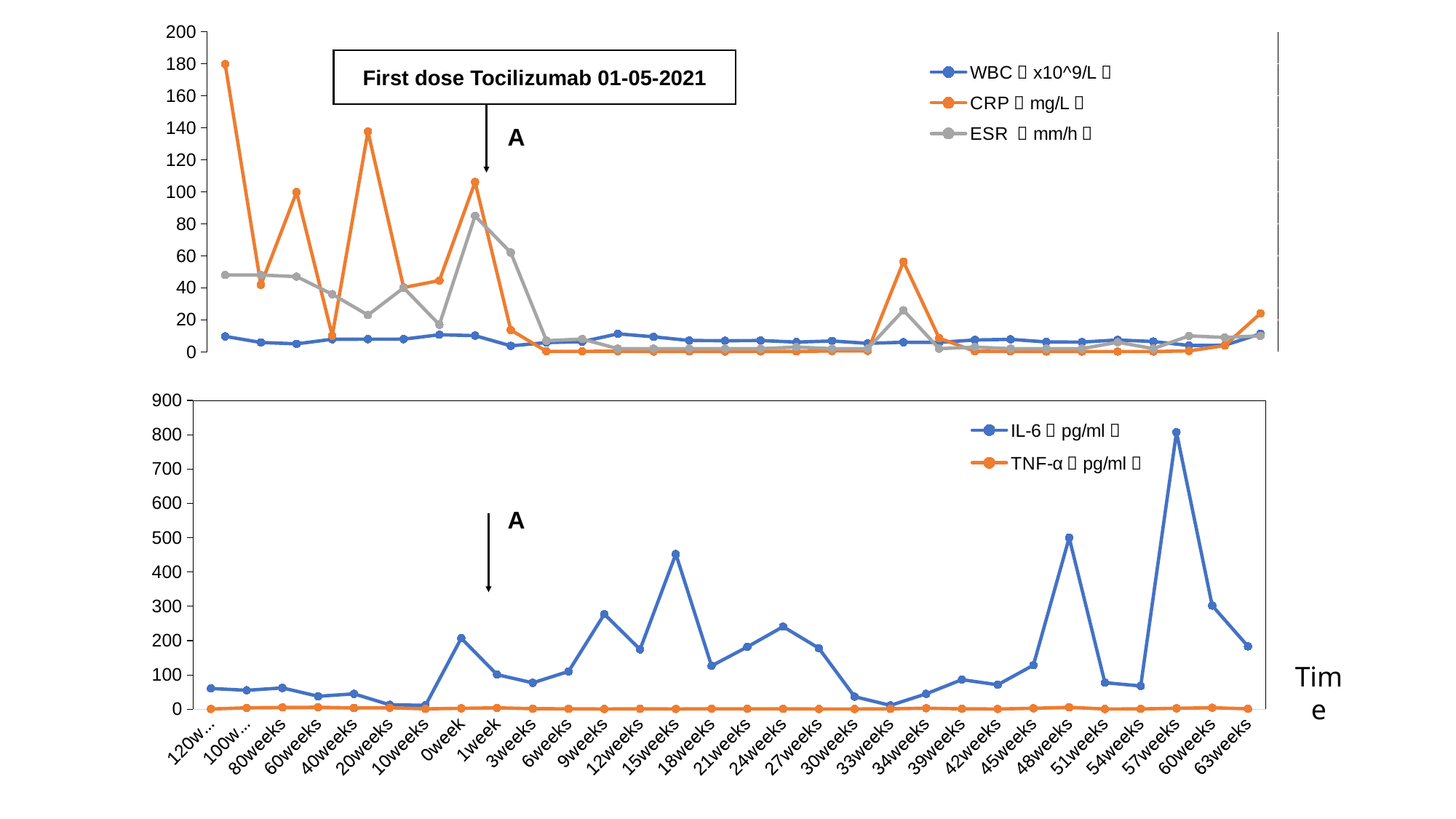

### Chart
| Category | WBC（x10^9/L） | CRP（mg/L） | ESR （mm/h） |
|---|---|---|---|
| 120week | 9.68 | 179.79 | 48.0 |
| 100week | 5.87 | 41.86 | 48.0 |
| 80week | 5.01 | 99.85 | 47.0 |
| 60week | 7.89 | 10.11 | 36.0 |
| 40week | 7.94 | 137.68 | 23.0 |
| 20week | 7.94 | 40.2 | 40.0 |
| 10week | 10.69 | 44.54 | 17.0 |
| 0week | 10.14 | 106.1 | 85.0 |
| 1week | 3.74 | 13.66 | 62.0 |
| 3week | 5.78 | 0.33 | 7.0 |
| 6week | 6.39 | 0.32 | 8.0 |
| 9week | 11.26 | 0.41 | 2.0 |
| 12week | 9.41 | 0.2 | 2.0 |
| 15week | 7.11 | 0.27 | 2.0 |
| 18week | 6.96 | 0.2 | 2.0 |
| 21week | 7.07 | 0.24 | 2.0 |
| 24week | 6.1 | 0.25 | 3.0 |
| 27week | 6.82 | 0.53 | 2.0 |
| 30week | 5.33 | 0.6 | 2.0 |
| 33week | 5.99 | 56.35 | 26.0 |
| 34week | 5.91 | 8.58 | 2.0 |
| 39week | 7.43 | 0.38 | 3.0 |
| 42week | 7.84 | 0.27 | 2.0 |
| 45week | 6.19 | 0.22 | 2.0 |
| 48week | 6.12 | 0.2 | 2.0 |
| 51week | 7.42 | 0.21 | 6.0 |
| 54week | 6.46 | 0.2 | 2.0 |
| 57week | 4.01 | 0.61 | 10.0 |
| 60week | 4.12 | 3.92 | 9.0 |
| 63week | 11.3 | 24.03 | 10.0 |First dose Tocilizumab 01-05-2021
A
### Chart
| Category | IL-6（pg/ml） | TNF-α（pg/ml） |
|---|---|---|
| 120weeks | 60.7 | 1.0 |
| 100weeks | 55.4 | 4.3 |
| 80weeks | 62.3 | 5.5 |
| 60weeks | 38.0 | 5.9 |
| 40weeks | 45.0 | 3.9 |
| 20weeks | 13.1 | 4.4 |
| 10weeks | 11.6 | 1.3 |
| 0week | 207.1 | 2.8 |
| 1week | 101.0 | 4.3 |
| 3weeks | 77.1 | 1.8 |
| 6weeks | 110.3 | 1.4 |
| 9weeks | 277.2 | 1.0 |
| 12weeks | 174.5 | 1.5 |
| 15weeks | 452.4 | 1.0 |
| 18weeks | 126.5 | 1.6 |
| 21weeks | 181.8 | 1.5 |
| 24weeks | 240.7 | 1.4 |
| 27weeks | 178.0 | 1.0 |
| 30weeks | 36.8 | 1.0 |
| 33weeks | 11.5 | 1.5 |
| 34weeks | 45.0 | 3.2 |
| 39weeks | 86.3 | 1.3 |
| 42weeks | 71.4 | 1.0 |
| 45weeks | 128.5 | 3.0 |
| 48weeks | 500.0 | 5.8 |
| 51weeks | 77.6 | 1.0 |
| 54weeks | 67.9 | 1.2 |
| 57weeks | 807.2 | 2.9 |
| 60weeks | 302.1 | 4.6 |
| 63weeks | 183.4 | 1.5 |A
Time

## Slide 4
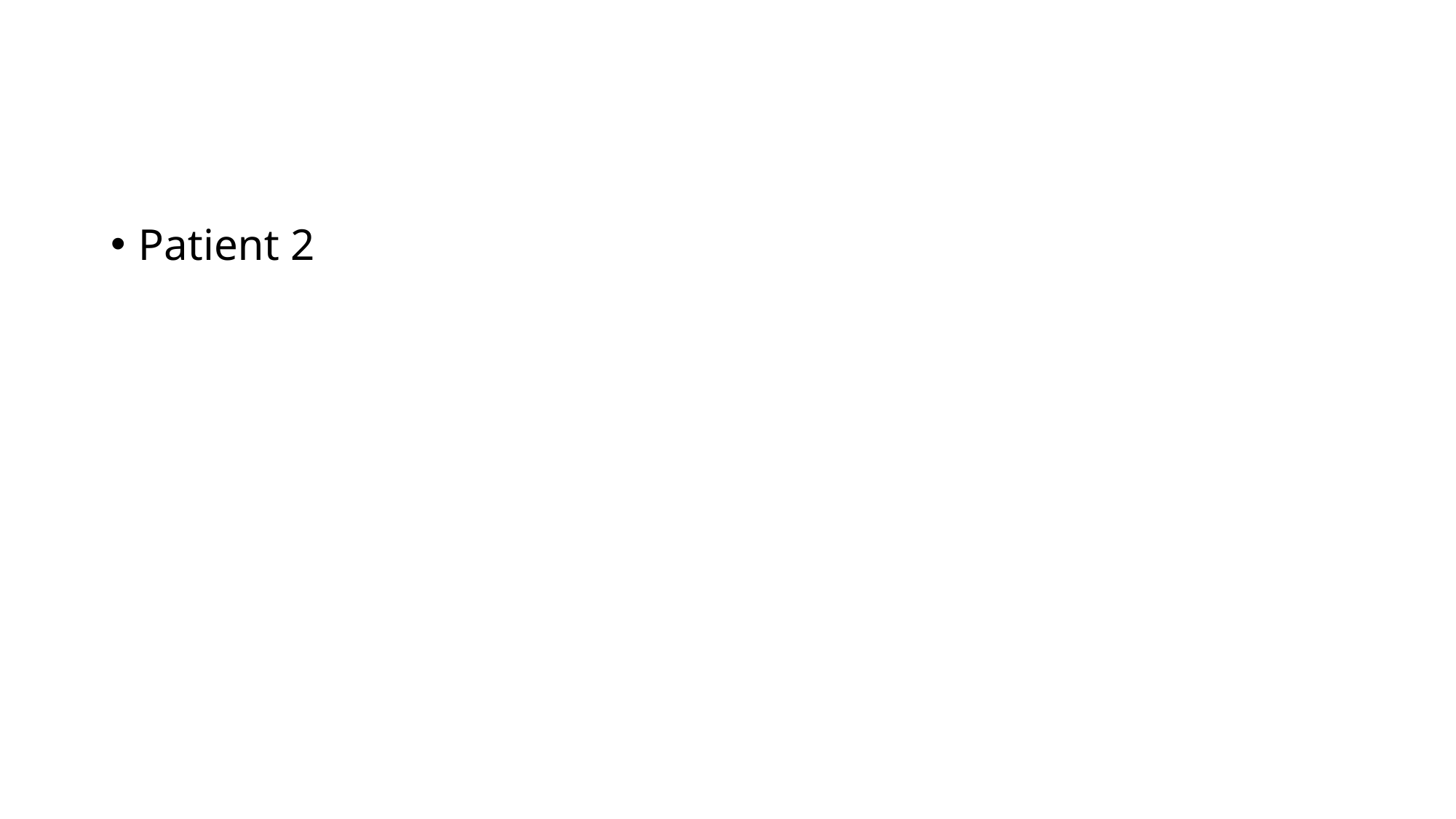

#
Patient 2

## Slide 5
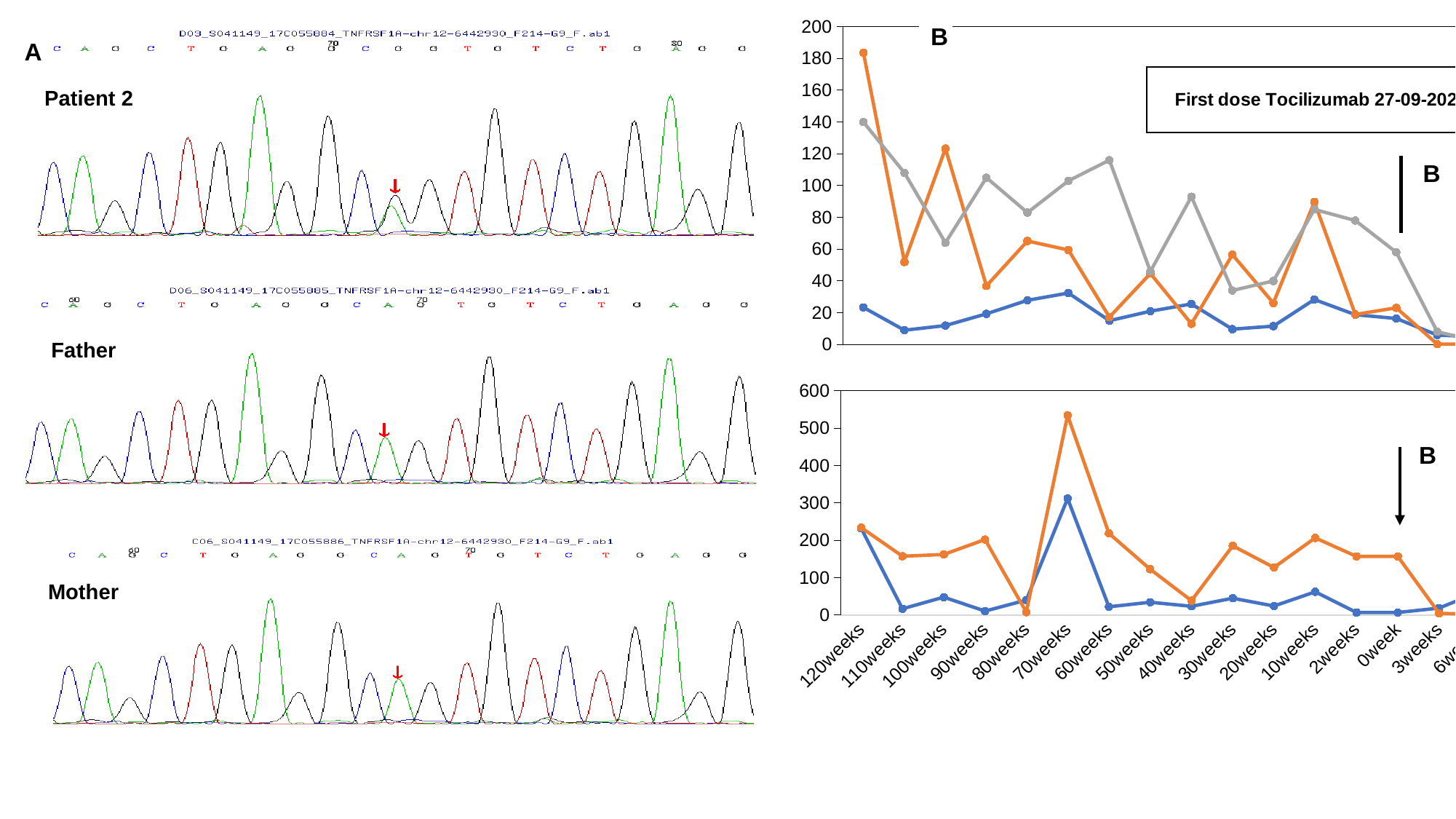

### Chart
| Category | WBC（x10^9/L） | CRP（mg/L） | ESR （mm/h） |
|---|---|---|---|B
A
Patient 2
Father
### Chart
| Category | IL-6（pg/ml） | TNF-α（pg/ml） |
|---|---|---|
| 120weeks | 232.1 | 234.1 |
| 110weeks | 16.8 | 157.5 |
| 100weeks | 47.9 | 162.2 |
| 90weeks | 10.6 | 202.2 |
| 80weeks | 40.1 | 7.8 |
| 70weeks | 312.0 | 534.6 |
| 60weeks | 22.2 | 218.9 |
| 50weeks | 34.3 | 122.8 |
| 40weeks | 23.5 | 38.6 |
| 30weeks | 45.0 | 185.4 |
| 20weeks | 24.2 | 127.5 |
| 10weeks | 62.2 | 206.6 |
| 2weeks | 6.8 | 157.1 |
| 0week | 6.8 | 157.1 |
| 3weeks | 18.7 | 4.7 |
| 6weeks | 63.7 | 1.7 |
| 9weeks | 23.5 | 2.3 |
| 12weeks | 4.5 | 1.5 |
| 15weeks | 11.2 | 2.1 |
| 18weeks | 20.1 | 3.1 |
| 21weeks | 21.1 | 1.5 |
| 24weeks | 20.6 | 2.0 |B
Mother
Time
