# Supplementary material for: Tocilizumab for treating mevalonate kinase deficiency and TNF receptor-associated periodic syndrome: a case series and literature review
Source: Pediatr Rheumatol Online J. 2024 Jan 5;22:11. doi: 10.1186/s12969-023-00952-2 (PMC10768362; doi:10.1186/s12969-023-00952-2)
Supplement: Supplementary file 5 — Supplementary Material 5 [file 12969_2023_952_MOESM5_ESM.docx]

Dear Editorial Officer:

This is Dr. Meiping Lu, the corresponding author of the manuscript titled “**Tocilizumab for Treating Mevalonate Kinase Deficiency and TNF Receptor-associated Periodic Syndrome: A Case Series and Literature Review**”. Our manuscript was previously submitted to this journal but was rejected due to some reasons. According to the reviewer's request, we have made revisions to the manuscript. The manuscript original title was “Tocilizumab for the Treatment of Mevalonate Kinase Deficiency and TNF Receptor-Associated Periodic Syndrome: cases report and literature review”（**Manuscript Number: PROJ-D-23-00105**）.

With the development of gene diagnosis, more and more SAIDs are recognized by pediatricians. Although different SAIDs have varying standard treatments, some SAIDs are poorly controlled after routine treatment, seriously affecting the growth and development of children and their quality of life. Tocilizumab could be an important addition to the treatment of SAIDs who were resistant to TNF-α blockade in our study. After more than half a year of follow-up observation, no serious adverse reactions were found. At present, more pediatric rheumatologists pay more attention to children's SAIDs, and my manuscript can provide more clinical support for pediatricians. The journal of Pediatric Rheumatology has good influence and credibility, so I hope the manuscript can been published on the journal.

All authors have read and approved the submission of the manuscript. The manuscript is being submitted only to ***Pediatric Rheumatology.*** The authors declare that we have no competing interests. All data and images have been approved by the Ethics Committee. We’d appreciate it if you could review and accept it.

Many thanks.

Best wishes,

Meiping Lu (Corresponding author, E-mail: meipinglu@zju.edu.cn)

Department of Rheumatology Immunology and Alllergy

Children’s Hospital, Zhejiang University School of Medicine, National Clinical Research Center for Child Health, Hangzhou 310052, China
