# Supplementary material for: Tocilizumab for treating mevalonate kinase deficiency and TNF receptor-associated periodic syndrome: a case series and literature review
Source: Pediatr Rheumatol Online J. 2024 Jan 5;22:11. doi: 10.1186/s12969-023-00952-2 (PMC10768362; doi:10.1186/s12969-023-00952-2)
Supplement: Supplementary file 2 — Supplementary Material 2 [file 12969_2023_952_MOESM2_ESM.doc]

**Response to reviewer's comments**

**Thank you for your careful review. It has played a great role in the improvement of our article. We deeply appreciate your valuable time. Should you have any other questions, please feel free to let us know kindly at any time. We have revised the manuscript according to your suggestions. All the changes of the manuscript have been marked red.**

Reviewer reports:

Reviewer #1: Manuscript Number: PROJ-D-23-00105
Title: Tocilizumab for the Treatment of Mevalonate Kinase Deficiency and TNF Receptor-Associated Periodic Syndrome: cases report and literature review
Pediatric Rheumatology

Reviewers' comments:
In this study, the authors reported two Chinese patients with MKD and TRAPS who have periodic fever and rash after using several drugs treatments. When they were treated with one or two doses of tocilizumab, they were not suffered from fever and rash. This treatment is lasted more than 6 months, and both of the patients have achieved serological remission without adverse reactions. The authors believed that tocilizumab as an alternative drug was useful for the treatment of SAIDs patients who were resistant to TNF-α blockade. While this manuscript is just a very simple report and literature review, it provides us some useful information on the MKD and TRAPS patients' treatment by using tocilizumab. Many questions should be addressed.

1. Higher definition images should be provided in the manuscript.
Response: Thanks. **The image we uploaded is very clear, and downloading it has affected the quality of the image. We can provide the original image.**

2. The statistical analysis of several clinical and serological parameters with previous reports or studies should be added for comparing the differences.
Response: Thanks. **White blood cells, C-reactive protein, and erythrocyte sedimentation rate can usually indicate disease activity in autoinflammatory diseases, so they have not been compared with previous reports or studies.**
3. Each gene mutation site in the referred genomes in Table 2 should be provided in detail.
Response: Thanks. **We have made modifications in Table 2 based on other literature and hope to meet the reviewer's requirements.**

4. Please declare the time of data presented in Table 1? And why do you choose the data in this time to show?
Response: Thanks. **The time of the data listed in Table 1 is the time when the two children were admitted to our hospital. Choosing the data at this time point for display is the baseline data of the two children before irregular treatment, which is of great significance.**
5. Since whether the gene mutations appeared in these two patients correlated with their diseases is not clear in this manuscript, the presentation of this content looks like lacking of necessary connections. If possible, please provide more evidence to support the correlation between gene mutations and MKD and TRAPS diseases.
Response: Thanks. **Both MKD and TRAPS gene mutation sites have been reported and are pathogenic.**

6. It is much better to label the increased and decreased clinical characteristics in these two patients compared with reference parameters.
Response: Thanks. **In our patients, the clinical symptoms and laboratory parameters of the patients have improved, as detailed in the manuscript.**

**After using TCZ, the temperature returned to normal, and the rash subsided gradually (Figures 1D and 1E). CRP and ESR returned to normal after six weeks. However, serum IL-6 level increased occasionally (Figure 2).**

**After three weeks of using TCZ, the temperature returned to normal, the rash subsided, and CRP, ESR, TNF-α, WBC, and IL-6 levels returned to normal (Figure 3B).**

7. The patients with MKD and TRAPS treated with tocilizumab looks good. However, the authors do not demonstrate whether they have other treatments when they have a tocilizumab injection. If the treatment effect of SAIDs is only relay on the tocilizumab treatment. Tocilizumab may very helpful for these kinds of patients in future. If not, it is hardly to say the tocilizumab treatment can have a very brilliant efficacy.
Response: Thanks. **Since there is no IL-1 related biological agent in Chinese Mainland, we have described in detail in this article that the symptoms of the patients with MKD and TRAPS are not well controlled after treatment of etanercept, baricitinib, glucocorticoids. So we chose tocilizumab to treat MKD and TRAPS.**

**Tocilizumab may very helpful for these kinds of patients in future.**

8. A lot of grammar and tense problems appeared in this manuscript. Probably the entire article should be improved with the help of a native-English speaker.
Response: Thanks. **We have revised the grammar and tense of the entire article.**

Reviewer #2:

1-Tocilizumab was beneficial to the treatment of MKD patients who were resistant to TNF-α blockade and IL-1 antagonists in case report (References needed, they're listed in the table but should be listed here too)

Response: Thanks. It changed into

According to previous case reports, tocilizumab (TCZ) is also beneficial in treating MKD patients who are resistant to TNF-α blockade [7-14].

7.	Rafiq NK, Lachmann H, Joensen F, Herlin T, Brogan PA. Tocilizumab for the Treatment of Mevalonate Kinase Deficiency. Case Rep Pediatr.2018;2018:3514645.

8.	Musters A, Tak PP, Baeten DL, Tas SW. Anti-interleukin 6 receptor therapy for hyper-IgD syndrome. BMJ Case Rep.2015;2015.

9.	Shendi HM, Devlin LA, Edgar JD. Interleukin 6 blockade for hyperimmunoglobulin D and periodic fever syndrome. J Clin Rheumatol.2014;20(2):103-5.

10.	Stoffels M, Jongekrijg J, Remijn T, Kok N, van der Meer JW, Simon A. TLR2/TLR4-dependent exaggerated cytokine production in hyperimmunoglobulinaemia D and periodic fever syndrome. Rheumatology (Oxford).2015;54(2):363-8.

11.	Lane T, Gillmore JD, Wechalekar AD, Hawkins PN, Lachmann HJ. Therapeutic blockade of interleukin-6 by tocilizumab in the management of AA amyloidosis and chronic inflammatory disorders: a case series and review of the literature. Clin Exp Rheumatol.2015;33(6 Suppl 94):S46-53.

12.	Rodrigues F, Philit J, Giurgea I, Anglicheau D, Roux J, Hoyeau N, et al. AA amyloidosis revealing mevalonate kinase deficiency: A report of 20 cases including two new French cases and a comprehensive review of literature. Seminars in Arthritis and Rheumatism.2020;50(6):1370-3.

13.	Lane T, Loeffler JM, Rowczenio DM, Gilbertson JA, Bybee A, Russell TL, et al. AA amyloidosis complicating the hereditary periodic fever syndromes. Arthritis Rheum.2013;65(4):1116-21.

14.	ter Haar NM, Jeyaratnam J, Lachmann HJ, Simon A, Brogan PA, Doglio M, et al. The Phenotype and Genotype of Mevalonate Kinase Deficiency: A Series of 114 Cases From the Eurofever Registry. Arthritis & Rheumatology.2016;68(11):2795-805.

2-Physical examination on admission revealed cervical, axillary and inguinal lymphadenectasis; abdominal softness, splenomegaly. (what is meant by lymphadenectasis and abdominal softness?)

Response: Thanks. **Lymph node enlargement is mainly to support the diagnosis of Kawasaki disease in the child, and the manuscript has been revised.**

It changed into

**On admission, physical examination revealed cervical lymph node enlargement and splenomegaly.**
3- For more than two years, the patient had bayberry tongue sometimes. (I'm not familiar with this terminology and internet search wasn't helpful. Can the authors explain what it means?_

Response: Thanks. **Bayberry tongue refers to** **oral mucous membrane changes in KD patients.**
4- In the patient, two pathogenic mutations were detected in the MVK gene (NM_000431.2): c.442G>A, p.Ala148Thr (A148T) and c.146T>A, p.Val49Glu(V49E) (Figure 1, C and F). At present, it is unclear whether MVK V49E is a pathogenic variant, which needs further verification by basic research. (The authors mention that 2 pathogenic variants were detected but later say that it is unclear whether the V49E variant is pathogenic. Which statement is correct? Also, what is meant by basic research? Functional testing?

Response: Thanks. **We are very sorry for the ambiguity caused by our carelessness, and it has been revised at the moment.**

It changed into

**In the patient, two pathogenic mutations were detected in the MVK gene (NM_000431.2): c.442G>A, p.Ala148Thr (A148T) and c.146T>A, p.Val49Glu(V49E).**

5- The level of IgD in serum was 200 mg/L and the MKD activity was 17.9 ug/L. The normal reference value range of plasma MVK activity of Chinese healthy children is 96.17±19.24 μg/L. (What is the normal range for serum IgD?)

Response: Thanks. **The normal range for serum IgD is 10-40mg/L.**

6-Providing a figure showing the timeline of events and treatment given would be helpful. Also, was barcitinib given only to the MVK patient? If so, that needs to be made clear.

Response: Thanks. **We agree with your statement. However, the patient's condition lasts for a long time, and their parents have sought medical treatment from multiple hospitals multiple times. The treatment is not standardized and it is difficult to provide specific treatment procedures.**

**Baricitinib was only given to the MVK patient.**

7- At last, we chose to inject it, 240mg each time (weight: 21 kg), every three weeks. (Providing a mg/kg dose would be easier for the reader).

Response: Thanks. It changed into

**Finally, we injected a dose of 12 mg/kg every three weeks.**

**On September 27, 2021, the boy began to take TCZ (dose: injection of 8 mg/kg once every three weeks).**

8- During the application of tocilizumab, there was an adenovirus infection with liver function damage. After treatment, the body temperature and liver function returned to normal without affecting the injection of tocilizumab. (This statement is bit unclear. Are the authors trying to mention that the patient developed an adenovirus infection that resolved without the need to withhold tocilizumab?)

Response: Thanks. **The purpose of this paragraph is to describe that the use of tocilizumab increases the chance of infection, but viral infection and liver function damage are mild and do not require discontinuation of medication.**
9- Anakinra inhibits not only IL-1β (and IL-1a) but also IL-6, which indicates a driving role for IL-1. (reference needed)

Response: Thanks. It changed into

In vitro and in vivo，anakinra inhibit not only IL-1β (and IL-1a) but also IL-6, indicating a driving role for IL-1 [10].

10.	Stoffels M, Jongekrijg J, Remijn T, Kok N, van der Meer JW, Simon A. TLR2/TLR4-dependent exaggerated cytokine production in hyperimmunoglobulin -aemia D and periodic fever syndrome. Rheumatology (Oxford).2015;54(2):363-8.

10-Consent and ethical approval is only listed under case 2.

Response: Thanks. **Both cases have passed the ethical application together.**

11-Table 2 formatting makes it very difficult to read.

Response: Thanks. **Table 2 contains a lot of content, and we have made appropriate modifications.**

12-Authors use wbcs, CRP, and ESR to monitor treatment response which is reasonable, but they should comment on the lack of reliability of a normal CRP while on IL-6 inhibiting treatment.

Response: Thanks. **Tocilizumab can inhibit inflammatory response and reduce CRP.**


Reviewer #3:

This is a case report on the use of tocilizumab for mevalonatkinase deficiency and TRAPS. The cases are interesting, but unfortunately the article is poorly written. The author(s?) should seek help from a native English speaking person. The report should also be condensed.

Response: Thanks. **We have revised the manuscript according to your suggestions, and streamlined the case reports.**

Is it one or two authors.? After the title only one is listed, but in the table immedeiatly below there are two

Response: Thanks. **Our manuscript has two authors, Yandie Li and Meiping Lu.**

In the Introduction the authors write that the systemic inflammatory diseases (SAID) are caused by mutations in genes coding for proteins in the innate immune system. This is only partially true - we have complex disorders also such as systemic JIA and PFAPA. Rewrite.

Response: Thanks. It changed into

**Systemic autoinflammatory diseases (SAIDs) are rare and severe diseases caused by gene mutations in innate immunity, leading to proinflammatory cytokine overproduction [1]. However, some SAIDs, such as systemic juvenile idiopathic arthritis (SJIA), are caused by multiple genes.**

Table 1 can be shortened and Table 2 is impossible to read. Also, there are two table 2 of which the last one is unnecessary and can be deleted.
Further evaluaton can only be done after the manuscript has been rewritten and shortened.
Response: Thanks. **We have revised the manuscript according to your suggestions, and streamlined the case reports.**

**Table 1 shows the parameters of two children before treatment with tocilizumab. We believe that table 2 and table 3 cannot be deleted. They are a literature review on the treatment of MKD and TRAPS with tocilizumab.**

**I am sorry for so many typos. We read the manuscript carefully again and corrected incomplete sentences and tables, and so on. I’d appreciate it if you could accept and review it again. I hope it could be considered for publication in future. I hope the manuscript can been published on the journal of *Pediatric Rheumatology*.**
